# Novel neural network classification of maternal fetal ultrasound planes through optimized feature selection

**DOI:** 10.1186/s12880-024-01453-8

**Published:** 2024-12-18

**Authors:** S. Rathika, K. Mahendran, H. Sudarsan, S. Vijay Ananth

**Affiliations:** 1 Prince Shri Venkateshwara Padmavathy Engineering College, Chennai, India; 2 Saveetha Engineering College, Chennai, India; 3 K. Ramakrishnan College of Engineering, Trichy, India; 4 Chennai Institute of Technology, Chennai, India

**Keywords:** Ultrasound Images, Fetal organs, Fast Radial Basis Function Neural Network, Optimization, Features, Accuracy, Data Balancing

## Abstract

Ultrasound (US) imaging is an essential diagnostic technique in prenatal care, enabling enhanced surveillance of fetal growth and development. Fetal ultrasonography standard planes are crucial for evaluating fetal development parameters and detecting abnormalities. Real-time imaging, low cost, non-invasiveness, and accessibility make US imaging indispensable in clinical practice. However, acquiring fetal US planes with correct fetal anatomical features is a difficult and time-consuming task, even for experienced sonographers. Medical imaging using AI shows promise for addressing current challenges. In response to this challenge, a Deep Learning (DL)-based automated categorization method for maternal fetal US planes are introduced to enhance detection efficiency and diagnosis accuracy. This paper presents a hybrid optimization technique for feature selection and introduces a novel Radial Basis Function Neural Network (RBFNN) for reliable maternal fetal US plane classification. A large dataset of maternal–fetal screening US images was collected from publicly available sources and categorized into six groups: the four fetal anatomical planes, the mother's cervix, and an additional category. Feature extraction is performed using Gray-Level Co-occurrence Matrix (GLCM), and optimization methods such as Particle Swarm Optimization (PSO), Grey Wolf Optimization (GWO), and a hybrid Particle Swarm Optimization and Grey Wolf Optimization (PSOGWO) approach are utilized to select the most relevant features. The optimized features from each algorithm are then input into both conventional and proposed DL models. Experimental results indicate that the proposed approach surpasses conventional DL models in performance. Furthermore, the proposed model is evaluated against previously published models, showcasing its superior classification accuracy. In conclusion, our proposed approach provides a solid foundation for automating the classification of fetal US planes, leveraging optimization and DL techniques to enhance prenatal diagnosis and care.

## Introduction

US imaging is a safe and effective method for monitoring the health of the baby and mother during pregnancy [1, 2]. It is non-invasive, offers real-time imaging capabilities, and is reasonably priced [3]. Commonly used during obstetric examinations, it helps achieve the best possible outcomes for both the mother and child. The most typical timing for a prenatal US is during the third trimester, typically between weeks 18 and 24 [4]. Obstetricians can measure and track fetal growth and development by capturing normal fetal planes during routine clinical examinations. Most countries provide prenatal care, which includes at least one scan in the third trimester to detect any anomalies. Fetal weight measurement is crucial for diagnosing anomalies and monitoring growth. Obstetricians calculate fetal weight using standard fetal US planes, which include four biometric parameters: abdominal circumference, femur length, biparietal diameter, and head circumference [5]. Maternal–fetal US images are frequently used to assess the health of the unborn child by evaluating biometric data such as gestational age and weight. The majority of fetal medicine institutes adhere to international standards established by scientific organizations when obtaining US images of both the mother and the unborn child. This ensures reproducible image acquisition using standardized approaches. Screening US performed in the middle trimester frequently provides more than 20 images per session. To enhance clinical evaluation, three-dimensional (3D) images, and films are sometimes acquired [6]. Fetal specialists select images that include structures of interest after analyzing the sonographer's findings. Confirmation by a senior maternal–fetal expert follows a team of certified research technicians' initial assessment. However, this procedure is challenging, error-prone, and time-consuming due to the large number of images in each screening US scan [7, 8]. Thus, a low-cost, presumably error-free automated system could perform the job. World Health Organisation (WHO) data indicate that sonographers in developing countries often lack the necessary education to conduct fetal US scans [9]. To overcome these challenges, automated classification of normal maternal–fetal US images can be implemented. This will aid inexperienced sonographers, reduce the burden of obstetricians, and increase diagnostic efficiency.

The recent decade has witnessed tremendous improvements in Artificial Intelligence (AI), particularly with the introduction of DL, and its success in image recognition tasks [10, 11]. DL has proven effective in various medical applications, including classifying and segmenting organs and lesions in computer tomography and magnetic resonance imaging images. These approaches excel at automatically detecting complex patterns in picture data and providing quantitative and qualitative assessments [12]. Early diagnosis of abnormalities relies on rapid and accurate assessment of US images to ensure the safety of both mother and fetus. This study aims to advance the field by presenting a hybrid optimized technique for better feature selection in maternal–fetal US images, as well as a fast RBFNN for accurate classification in this domain.

Gynecologists utilize US images to detect pregnancy problems and monitor the progress of a developing fetus. US is widely employed in the medical field for categorizing fetal standard planes. This article describes a unique methodology for detecting maternal–fetal planes in US images based on the RBFNN architecture. RBFNNs are a popular type of neural network used in computer vision and image processing. The primary contributions of this study are as follows:

1. Maternal fetal US images were collected from a large publicly accessible dataset.
2. Several preprocessing techniques are implemented, such as scaling, removing undesirable portions from US images, improving image quality, and extracting features.
3. Use the hybrid optimized PSOGWO method to choose the most significant features, enhancing the classifier's accuracy.
4. The novel DL model, Fast-RBFNN is proposed with ε-insensitive loss function and structural risk term. This technique not only maintains robust nonlinear fitting with easy learning rules, but it also handles huge data sets quickly and efficiently.
5. Evaluate the proposed DL model against conventional DL models such as Artificial Neural Network (ANN), Convolutional Neural Network (CNN), and Radial Basis Function (RBF). Additionally, a comparison with current works is performed.

The study's framework is presented below. The "Related work" section includes an overview of research associated with this domain. The "Materials and Methods" section details data collection, processing, feature extraction, selection, and classifiers. The outcome of the proposed model is discussed in the "Results and Discussion" section. Finally, the "Conclusion" section discusses the effectiveness of the proposed methodology and potential areas for future research.

## Literature survey

AI systems' predicting and classifying skills are driving wider applications in clinical research. As a result, they have found widespread application in biomedical research and the development of reliable tools for diagnosis. Numerous studies have been conducted to classify maternal–fetal standard planes using ultrasonography.

Image categorization, facial recognition, and speech recognition are just a handful of the challenges that AI has helped overcome. However, this strategy will not be very effective for classifying and segmenting US images. The automatic categorization of anatomic planes from fetal US images is complicated by several issues, including poor SNR, the mother's bulk, and the small size of the fetus [13]. The problems are made worse by the fact that images in hospital archives are taken with a range of equipment types, operators, imaging formats, resolutions, zoom settings, and so forth. Various DL models like GoogLeNet [14] and ResNet [15] have been proposed to overcome the above challenges. The use of these algorithms in classification tasks involving medical images yields excellent results. The study [16] used Deep Convolutional Neural Networks (DCNNs) to increase recognition accuracy and optimize clinical pipelines by identifying fetal facial standard planes (FFSPs). Training DCNN models with improperly labeled examples caused overfitting and performance loss. The study [17] proposed a Transfer Learning (TL) strategy to overcome the issues of limited training data and diminishing performance. This method involves implanting the knowledge of specific CNN models that have been pre-trained on a broad scale with images of natural settings. These models are used to localize medical images such as fetal abdominal standard planes. When it came to the auxiliary task of plane recognition, the metrics measurements demonstrated that DCNN and TL were effective. The algorithm's limitations in real-time manipulation and reliance on quantitative medical datasets rendered it unsuitable for practical usage. Recent works [18] propose a unique 82-layer DL architecture based on residual bottleneck mechanisms. The intended design now includes three more blocks, each with highway routes and skip connections. Furthermore, before each residual block, a convolutional layer has been added. During training, several hyperparameters were initialized using Bayesian optimization (BO). The classification is performed with deep features taken from the average pooling layer. A more efficient Moth Flame Optimization strategy for feature selection was proposed to address increased computation time during the classification phase. Next, the selected features are used to train neural network classifiers to categorize the data. During the experimental phase, US images were evaluated, with a focus on normal mother-fetal images and images of the fetus's developing brain. Some limitations are (i) A bias in the dataset complicates DL model training; and (ii) the deep layer extracts irrelevant data.

The research [19] aims to improve fetal brain US plane categorization by investigating and evaluating the use of a Generative Adversarial Network (GAN) to generate synthetic US images of the brain. The synthesis of fetal brain images utilizing cutting-edge GANs stylegan2-ada was compared to baseline classifiers that used GAN-based data augmentation. Our experiments suggest that combining GAN-generated data with standard augmentation techniques improves accuracy. The focus was solely on binary categorization, ignoring all other possible options. Using a brain detector, the US images of the developing brain were pre-processed and centered. No evaluation was conducted on these architectures' performance on raw US images. The paper [20] proposed a novel framework called FetalBrainAwareNet for generating anatomically accurate synthetic images of fetal head standard planes (FHSPs). This framework enhances the presence of desired anatomical features in the generated images. It also explores specific regularization factors in the adversarial training loss function to control the fetal skull shape and improve the distinction of standard planes, ensuring that the synthetic images structurally and visually match real US scans. The framework demonstrates its versatility by generating high-quality images of the three most common FHSPs. Quantitative and qualitative results indicate that the system produces a greater variety of US images compared to existing methodologies. In the article [21], a new U-Net-based network for medical image segmentation was developed. This enabled us to approach the problem. The architecture of the proposed model consists of four layers: two-dimensional convolutional, two-dimensional transposed convolutional, and batch normalization. The encoder-decoder path consists of four blocks. The performance of the proposed network was tested on a publicly available database for fetal head circumference estimation and a newly created database for head and belly circumference. The suggested fast network significantly reduced processing time when compared to existing U-Nets. The suggested model improved segmentation accuracy since it has more trainable parameters than comparable models. The researchers [22] present a method for automatically identifying 14 distinct fetal characteristics in 2-dimensional fetal US images by integrating data from the entire image and selected areas of interest (AOI). Our solution uses pre-trained CNNs to learn two feature extractors on entire as well as AOI of US fetal images. Our approach is unique in that it combines classification decisions based on global and local features without the usage of prior knowledge. Furthermore, our method can identify fetal features in the image based on the categorization results. In comparison to the other non-fusion-based techniques, the proposed solution is statistically significantly better. Because of this limitation, the developed AI algorithms may be less effective or generalizable, and they will be unable to handle a broader range of clinical circumstances.

To improve the accuracy of classical ultrasonic plane detection, graph-enhanced multi-scale structure perception architectures were used [23]. Specifically, a graph-based multi-view refinement module was introduced for detecting linkages between fetal anatomical components, along with a local-to-global multi-granularity ensemble module for feature enhancement and noise suppression. A confidence assessment loss was added, and several classifiers with branch subnets were employed to produce fine-grained structure representations. Confidence matching enables classifiers to collaborate while generating choices. The paper [24] proposes an automated method for recognizing common fetal US planes using a CNN stacking ensemble. This strategy employs three CNNs: AlexNet, VGG-19, and DarkNet-19. Predictions from these CNNs are extracted using random forest and softmax classifiers. The final forecast is determined using the absolute majority voting method. The stacking ensemble method was evaluated on a freely available prenatal US dataset, showing superior performance compared to competing approaches and individual CNN models. The study [25] explores the effectiveness of SimCLR in scenarios with low and high inter-class variability, considering that classification performance varies with the number of labels. Various training approaches utilizing contrastive learning were employed, alongside quantitative and qualitative research using industry-standard measurements. Contrastive learning proved to be more effective for low inter-class variability classification tasks, particularly when trained with ImageNet weights, compared to high inter-class variability tasks.

The study [13] proposes an automated approach for identifying regularly utilized fetal US planes using the DCNN stacking ensemble. All three DCNNs have been pre-trained using the stacking ensemble technique. Random forest and softmax classification algorithms are utilized to make predictions for DCNNs. The final forecast is generated with the majority vote method. The stacking ensemble method is evaluated on a freely available fetal US dataset. The suggested ensemble model for fetal US planes establishes six categories in total. The experiment results reveal that the stacking ensemble method was extremely effective. Training DCNN models with improperly labeled examples caused overfitting and performance loss. Article [24] describes a DL-based automated categorization system for fetal US planes to improve detection efficiency and accuracy. The suggested solution is mostly made up of feature integration and categorization modules. Following the retrieval of deep features using TL models, the global average pooling layer is first merged. Merging deep features from multiple CNNs produces a more robust feature representation. The next stage is to employ a multi-layer perceptron with deep features to predict fetal US images. When compared to other cutting-edge models that are already available, the proposed technique demonstrated higher categorization efficiency. This method's possible downsides include difficulty with real-time use, overfitting, model generalization, and dataset quality [26].

In existing research, many DL models have been developed, but they still struggle to achieve excellent accuracy in classifying fetal organs from US images. These models can be adversely affected by noise present in medical images, and some fail to extract important features from US images effectively. Feature extraction is crucial for obtaining better results. Additionally, some models suffer from overfitting problems. To address these issues, this research presents a hybrid PSOGWO technique for effective feature selection and proposes Fast-RBF with an ε-ILF and structural risk minimization to avoid overfitting.

## Proposed methodology

Publicly accessible US image data containing maternal–fetal US planes are gathered for analysis. Raw US images undergo careful pre-processing to enhance their quality and suitability for classification purposes. The pre-processing steps are detailed in Section III.B. GLCM is employed to extract features, that are effective for texture analysis—a crucial aspect in US imaging where texture patterns provide significant insights into the anatomical structures of fetal planes. GLCM extracts 45 distinct features from each image, capturing essential texture information for classification. To improve classification accuracy and speed, optimization techniques are utilized to reduce the feature dimension. The PSO algorithm is well-known for its ability to solve a variety of real-world issues. However, PSO might get caught in local minima. To address this problem, the GWO is employed to assist PSO, preventing local minima and improving the search process. PSO, GWO, and their hybrid, PSOGWO, are used to identify the most important features. Through this process, the original set of 45 features is reduced to 25.

**Fig. 1:**
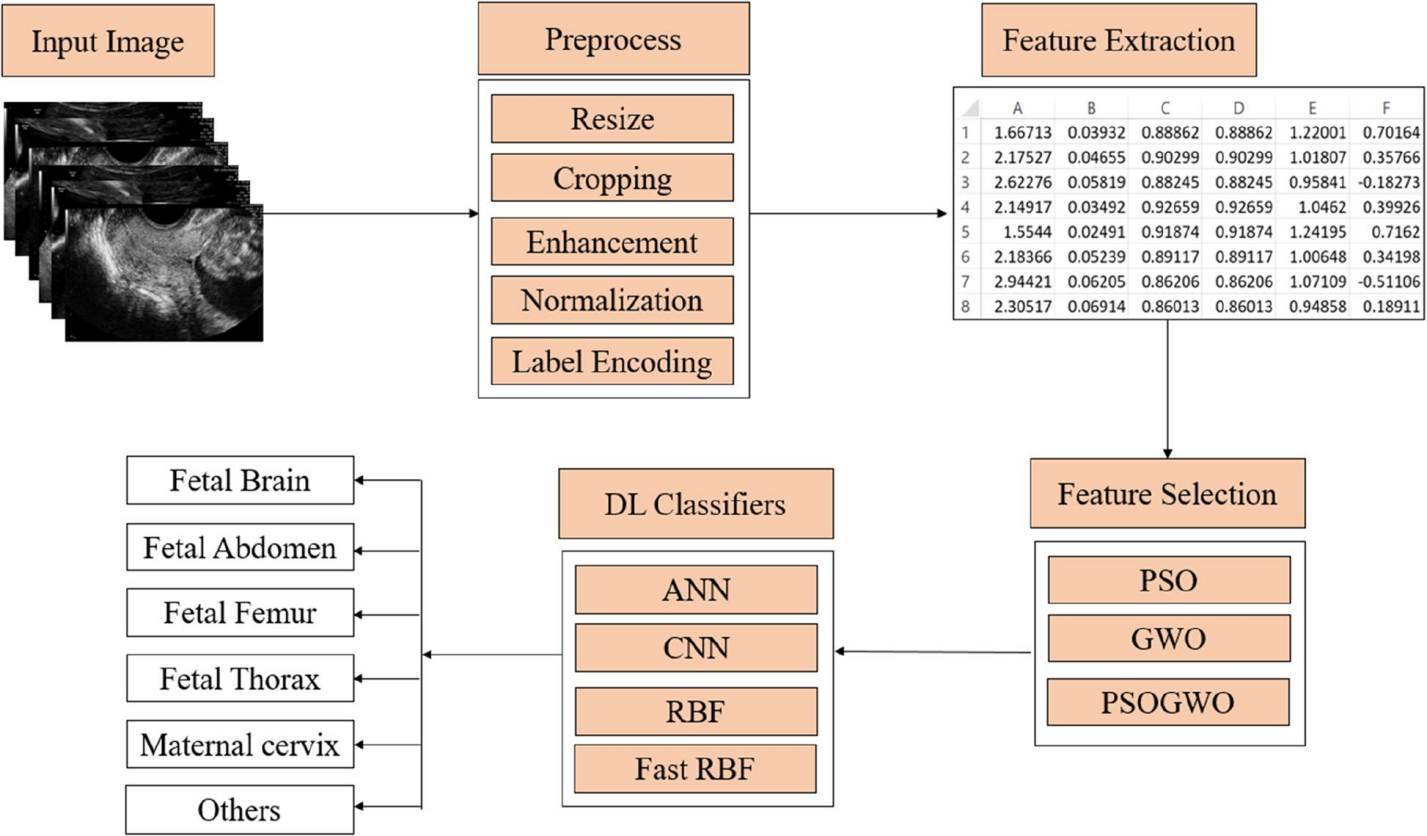
Proposed methodology workflow

The selected features from each optimization algorithm are inputted into various DL models, including ANN, CNN, RBF, and the proposed DL model. The performance of each combination of the feature selection algorithm and DL model is evaluated using appropriate metrics. The model achieving the highest classification performance based on the evaluation metrics is identified as the best classifier for maternal fetal US planes. The proposed methodology for maternal fetal classification is visually represented in Fig. 1.

### Data acquisition

**Fig. 2:**
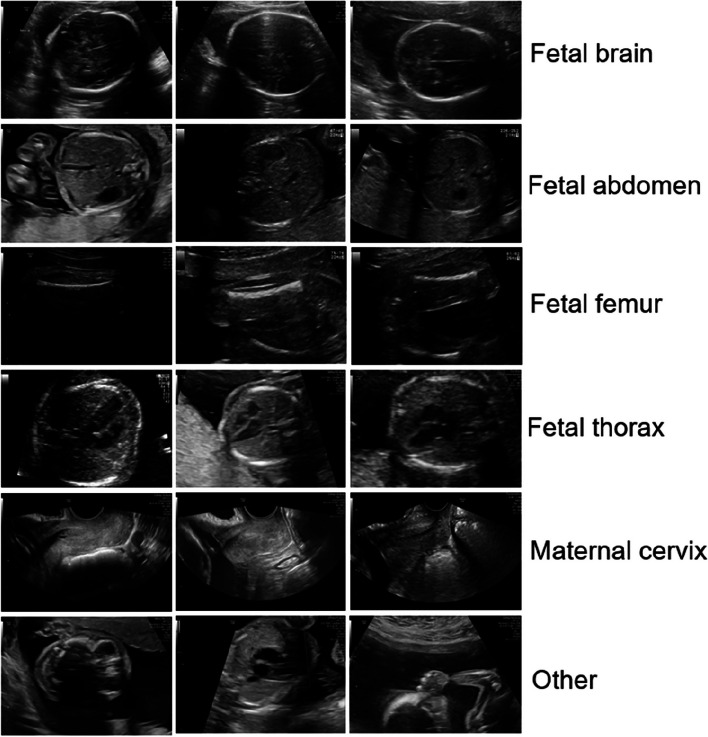
Sample US images from the Zenodo dataset

The fetal dataset from Zenodo [27] was used for this study. The collection consists of 12,400 US pictures. Images were divided into five anatomical planes (brain, abdomen, femur, thorax, and maternal cervix) that are most commonly used for maternal–fetal screening, as well as an "Other" category that includes additional planes. Figure 2 shows samples of US images from each category. The details of images are listed in Table 2. According to this table, the number of images in each type ranges from 714 to 4,213. As a result, it appears that there is an issue with class imbalance.

### Data processing

To learn image-based AI models, images need to be first processed. In the absence of pre-processing, Low-quality input data might cause AI models to underperform. Several methods are employed in pre-processing techniques to improve the input data, such as resizing, cropping, noise removal, image enhancement, balancing, and encoding.

Variations in image size in raw US images can impair model accuracy [28]. Images are scaled down to a standard resolution while maintaining their aspect ratio to ensure consistency and save computing load. The image resize process can be described in Eq. (1):

1 $$Resized\;Image=resize\;(Original\;Image,\;Target\;Resolution)$$

where the raw US image is referred to as the $Original Image$. The $Target Resolution$ specifies the ideal image resolution to be achieved when resizing.

Cropping is a typical technique for focusing on a specific part of an image and removing distracting background components [29]. There is a lot of irrelevant information (patient data, system settings, image position, etc.) in the provided dataset that ruins the images' ability to show the AOI. Model reliability and image readability are improved by cropping. It is a simple and effective strategy for improving image quality, resulting in more relevant and legible data for the suggested models. By reducing superfluous data, our cropping method improves model accuracy and speeds up training. When cropping an image, the height $(h)$ and width $(w)$ of the edges are first measured. The cropping borders are then set, ensuring that the x and y values remain within permissible limits. The cropping method uses the computed $x$ and $y$ values, as well as $h$ and $w$ values.

Image processing techniques are used to improve the contrast of US images and uncover previously concealed elements. The term "histogram equalization," is in Eq. (2):

2 $$Enhanced\;Image=histeq\;(Croped\;Image)$$

Here, the image after cropping is termed as $Croped Image$. The histogram equalization operation is represented by $histeq$.

Normalizing pixel values to a constant range is essential for reliable model training. Normalizing pixel values to the [0, 1] range is a common practice in image processing [30]. Each image has an individual number that corresponds to the name of the anatomical plane associated with it. Since most ML approaches require numerical inputs. The outcome of the pre-processed US image at each stage is given in Table 1.

**Table 1:**
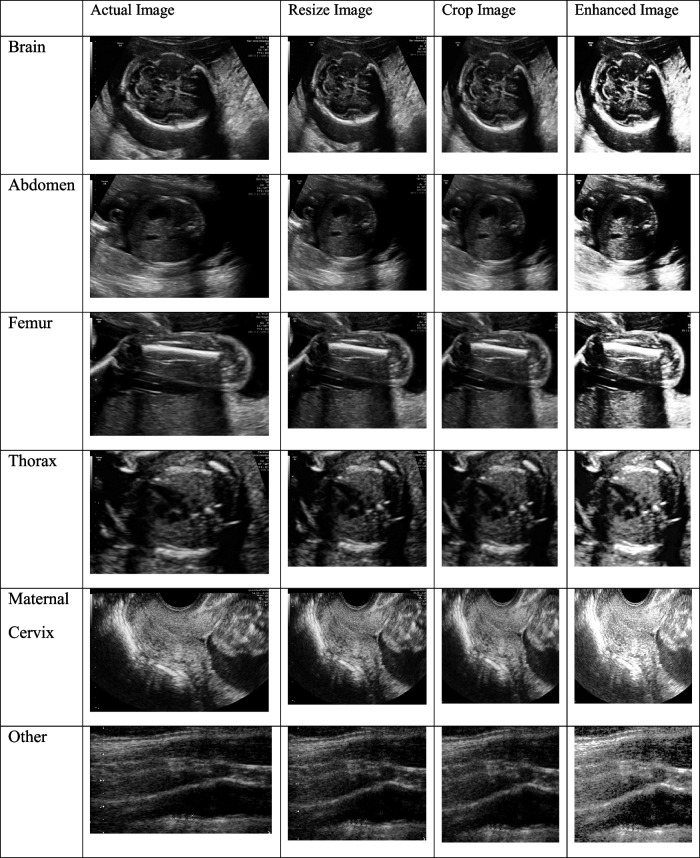
Pre-processed image outcome at each stage

**Fig. 3:**
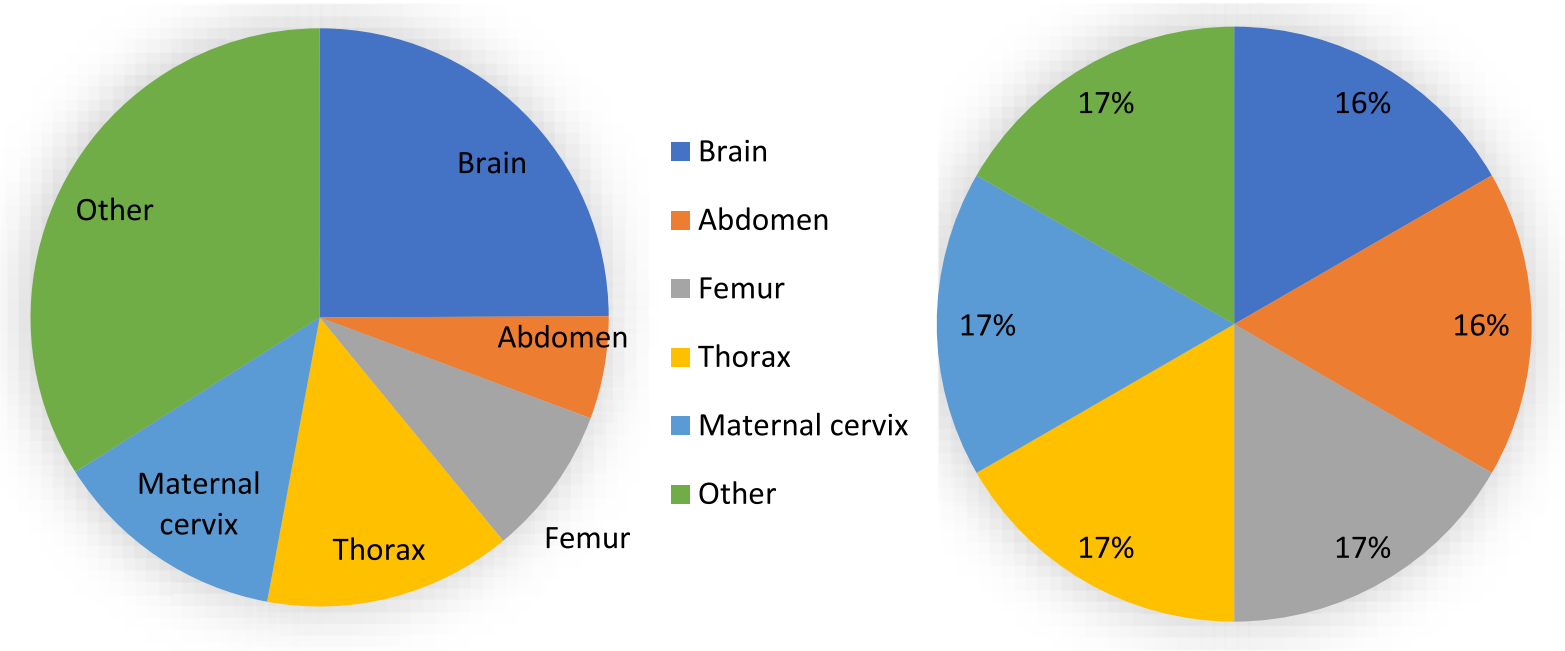
Distribution percentage of US images before and after sampling

Data augmentation approaches were employed to address class imbalance in the dataset's minority classes. Oversampling and undersampling techniques [31] were used to overcome this issue, as shown in Table 2. This strategy involved boosting and reducing the representation of minority and majority classes by randomly removing some cases and duplicating existing samples. Each group has been allotted an instance of 1050. This strategy, which distributes the dataset's classes more evenly, can improve the model's accuracy. Figure 3 depicts the US image distribution before and after applying the sampling approaches. This method significantly improves the effectiveness of the proposed models by rectifying the class imbalance [32].

**Table 2:** US fetal image distribution

Classification	Actual	After balancing
Fetal Brain	3092	1050
Fetal Abdomen	711	1050
Fetal Femur	1040	1050
Fetal Thorax	1718	1050
Maternal Cervix	1626	1050
Other	4213	1050

### Feature extraction

The texture is created by spatially alternating grayscale values, resulting in a spatial link between two pixels in the image separated by a set distance. An accurate approach to texture description is examining the spatial connection of grayscale values. The GLCM is a popular technique for showing the spatial correlation of pixel grayscale [33]. It characterizes a picture by taking into account the distance between adjacent pixels, the magnitude of variation, and the direction of correlation. The essential notion is to compute the frequency of occurrence of two grayscale pixels in a certain spatial arrangement, which can be used to assess the image's regional relativity and consistency. As the distance changes, GLCM evolves immediately in fine textures but slowly in coarse textures. The GLCM defines a square matrix whose magnitude corresponds to the likelihood that a gray value $g1$ is separated from another gray value $g2$ by a fixed spatial location connection (dimensions and orientation). Consider $f(i,j)$ as a two-dimensional grayscale image, where $S$ is the collection of pixels within the region that have a certain spatial relationship and $P$ represents the GLCM, which is expressed in Eq. (3)

3 $$P\left(i,j\right)=\frac{\#\left\{\left[\left({i}_{1},{j}_{1}\right),\left({i}_{2},{j}_{2}\right)\right]\in S|f\left({i}_{1},{j}_{1}\right)={g}_{1 }\& f\left({i}_{2},{j}_{2}\right)={g}_{2}\right\}}{\#S}$$

There are two techniques to obtain pixel information from GLCM: distance and angle orientation [34]. When the distance is too small, the data in each pixel becomes extremely constant. However, going overboard renders the data between pixels worthless. The distance d = 5, and the scenario encompasses four angles: {${0}^{^\circ },{45}^{^\circ },{90}^{^\circ } and {135}^{^\circ }$}. The scenario applies to all patch images due to the presence of multipatch images. Feature extraction using GLCM retrieves data associated with specific variables, including energy, correlation, dissimilarity, homogeneity, and contrast. After converting RGB images to grayscale, GLCM extracts pixel information. GLCM utilizes two orientations: angle and distance. The co-occurrence matrix will be updated before the GLCM variable is computed. Divide the total number of recorded co-occurrences by the specified normalization. As previously stated, the variables will be calculated after normalization. This is a reference to the Haralick publications, which were critical to the development of GLCM [35]. Each of the variables is defined in Eq. (4–13).

4 $$Contrast=\sum_{i,j=0}^{{N}_{g-1}}p\left(i,j\right){\left(i-j\right)}^{2}$$

5 $$Dissimilarity=\sum_{i,j=0}^{{N}_{g-1}}p\left(i,j\right)\left|i-j\right|$$

6 $$Homogenity=\sum_{i=1}^{{N}_{g}}\sum_{j=1}^{{N}_{g}}\frac{p\left(i,j\right)}{1+{\left(i-j\right)}^{2}}$$

7 $$Energy=\sum_{i}\sum_{j}{\left\{p\left(i,j\right)\right\}}^{2}$$

8 $$ASM=\sum_{i}\sum_{j}{\left\{p\left(i,j\right)\right\}}^{4}$$

9 $$Correlation=\frac{\sum_{i=1}^{{N}_{g}}\sum_{j=1}^{{N}_{g}}\left[\left(ij\right)p\left(i,j\right)\right]-{\mu }_{x}{\mu }_{y}}{{\sigma }_{x}{\sigma }_{y}}$$

10 $$Entropy=-\sum_{i}^{N}\sum_{j}^{N}P\left(i,j\right)lg P\left(i,j\right)$$

11 $$Mean={\mu }_{i}=\sum_{i,j=0}^{N-1}i\left({P}_{i,j}\right) , { \mu }_{j}=\sum_{i,j=0}^{N-1}j\left({P}_{i,j}\right)$$

12 $$Variance= {\sigma }_{i}^{2}=\sum_{i,j=0}^{N-1}{P}_{i,j}{\left(i-{\mu }_{i}\right)}^{2}, { \sigma }_{j}^{2}=\sum_{i,j=0}^{N-1}{P}_{i,j}{\left(j-{\mu }_{j}\right)}^{2}$$

13 $$Standard Deviation= {\sigma }_{i}=\sqrt{{\sigma }_{i}^{2}}, {\sigma }_{j}=\sqrt{{\sigma }_{j}^{2}}$$

The GLCM co-occurrence matrix element $p\left(i,j\right)$ is normalized and symmetric in relation to ${\left(i,j\right)}^{th}$. Contrast is defined as the intensity of the pixels surrounding the reference pixel at a given angle and distance. In general, great contrast indicates a lively image. Dissimilarity will calculate the spacing between any two items in the area of interest, as represented by pixels. A higher value implies that there is a considerable difference in intensity between adjacent pixels. The homogeneity of a pixel's distribution in a GLCM is measured. The relationship between contrast and value will be inverse. The contrast decreases as the degree of homogeneity increases. ASM measures the regularity of pixels in GLCM. The higher the ASM number, the more similar the pixels are. ASM provides 100% of GLCM's power. ASM's base is energy. The correlation demonstrates a local gray-level dependency on the texture picture through the linear dependency of the gray-level value in the GLCM. A comparable gray-level area can produce stronger correlation results. The ultimate purpose of all of the equations is to supply six texture features per image patch to train the deep neural network. All four of these angles $\left({0}^{^\circ },{30}^{^\circ },{45}^{^\circ },{90}^{^\circ } and {135}^{^\circ }\right)$ represent the location of the focus pixels. Meanwhile, the distance between the reference and nearby pixels is $d = 5$. The position is determined first, and then each variable is calculated. A total of 45 features were collected from GLCM. The hyperparameters used in this study are detailed in Table 3.

**Table 3:** GLCM Hyperparameters

Hyperparameter	Values
Patches	1000
Distance	5
Angles	${0}^{^\circ },{30}^{^\circ },{45}^{^\circ },{90}^{^\circ } and {135}^{^\circ }$
Number of Features	45

### Feature selection

#### PSO

In the research [36], the PSO algorithm was used for feature selection in COVID detection and achieved better results. Based on this, we decided to choose PSO. The algorithm 1 demonstrates how PSO represents the evolution of understanding of social behavior and the dynamics of group communication during the exchange of secret information regarding migration, flocking, or hunting [37]. Together, they form a solution; the former is called a swarm, while the latter is called particles. Particles can modify their positions by utilizing both their own and the information of their neighbors [38].


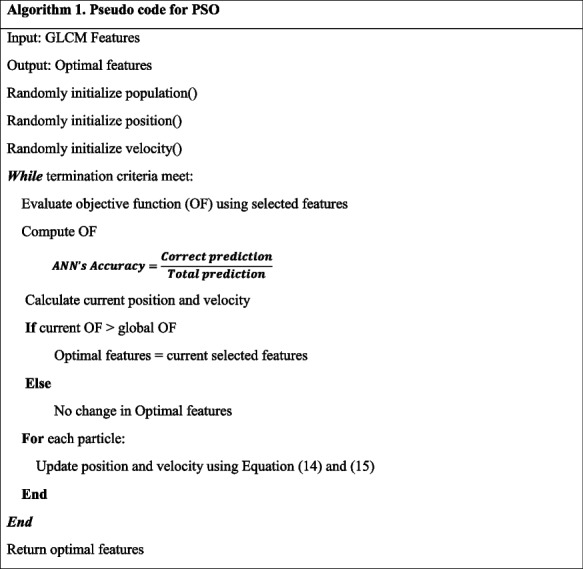


To begin, the swarm generates a set of random particles dependent on positions and velocities. The procedure for updating the particles' locations and velocity is represented by Eqs. (14) and (15):

14 $${x}_{ij}^{\left(t+1\right)}={x}_{ij}^{\left(t\right)}+{v}_{ij}^{\left(t+1\right)}$$

15 $${v}_{ij}^{\left(t+1\right)}={wv}_{ij}^{\left(t\right)}+c1r1\left({x}_{ij}^{p\left(t\right)}-{x}_{ij}^{\left(t\right)}\right)+c2r2\left({x}_{ij}^{g\left(t\right)}-{x}_{ij}^{t}\right)$$

Here, $t$ represents the current iteration and $w$ is an inertia weight, used to expedite population convergence. When the position of the $i$-th particle in the $j$-th dimension is denoted by ${x}_{ij}$ and its velocity by ${v}_{ij}$. Additionally, $c1$ and $c2$ are acceleration coefficients, constant values. The previous best position of particle $i$ in the $j$-th dimension is represented by ${x}_{ij}^{p\left(t\right)}$ and ${x}_{ij}^{g\left(t\right)}$ respectively. The parameters $r1$ and $r2$ can take values between 0 and 1. Then, the main loop of PSO evaluates each particle using a fitness function and compares the results with both local and global best values.

#### GWO

The GWO algorithm simulates the leadership structure and hunting strategies of grey wolves [39]. The alpha (α), beta (β), delta ($\updelta$), and omega (Ω) packs of grey wolves serve as a model for the organizational structure. Additionally, the algorithm incorporates the three main components of hunting: seeking out prey, encircling prey, and attacking prey [40]. Algorithm 2 presents the pseudocode for the GWO algorithm.


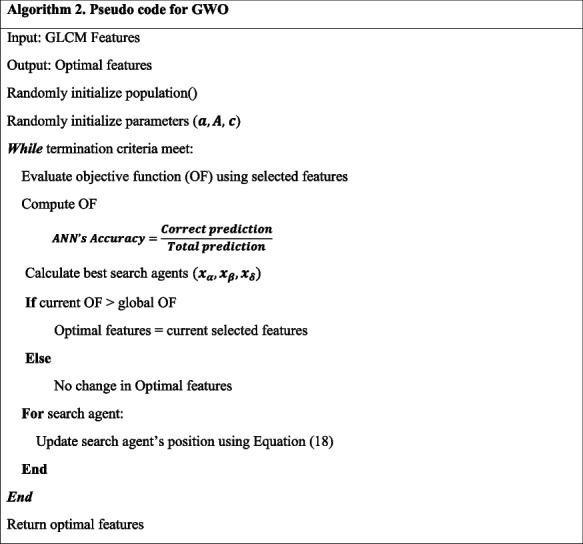


Here is a brief rundown of the grey wolf hunting method: The α, β, and $\updelta$ are likely the most knowledgeable about the potential whereabouts of prey. Alpha represents the best candidate solution. By retaining the top three results, it can be ensured that all search agents, including the omegas, will be compelled to adjust their positions to match those of the top search agents. Grey wolves use these formulas from Eq. (16–18) to keep track of their positions.

16 $${D}_{\alpha }=\left|\overrightarrow{{c}_{1}}.\overrightarrow{{x}_{\alpha }}-\overrightarrow{x}\right|, {D}_{\beta }=\left|\overrightarrow{{c}_{2}}.\overrightarrow{{x}_{\beta }}-\overrightarrow{x}\right|, {D}_{\delta }=\overrightarrow{{c}_{3}}.\overrightarrow{{x}_{\delta }}-\overrightarrow{x}$$

17 $$\overrightarrow{{x}_{1}}=\overrightarrow{{x}_{\alpha }}-\overrightarrow{{A}_{1}} . \overrightarrow{{D}_{\alpha }}, \overrightarrow{{x}_{2}}=\overrightarrow{{x}_{\beta }}-\overrightarrow{{A}_{2}} . \overrightarrow{{D}_{\beta }}, \overrightarrow{{x}_{3}}=\overrightarrow{{x}_{\delta }}-\overrightarrow{{A}_{3}} . \overrightarrow{{D}_{3}}$$

18 $$\overrightarrow{x\left(t+1\right)}=\frac{\overrightarrow{{x}_{1}}+ \overrightarrow{{x}_{2}}+\overrightarrow{{x}_{3}}}{3}$$

The distances between each $\delta , \beta$, and $\alpha$ and the prey are represented by $\overrightarrow{{x}_{1}}$,$\overrightarrow{{x}_{2}}$, and $\overrightarrow{{x}_{3}}$.

#### PSOGWO

**Fig. 4:**
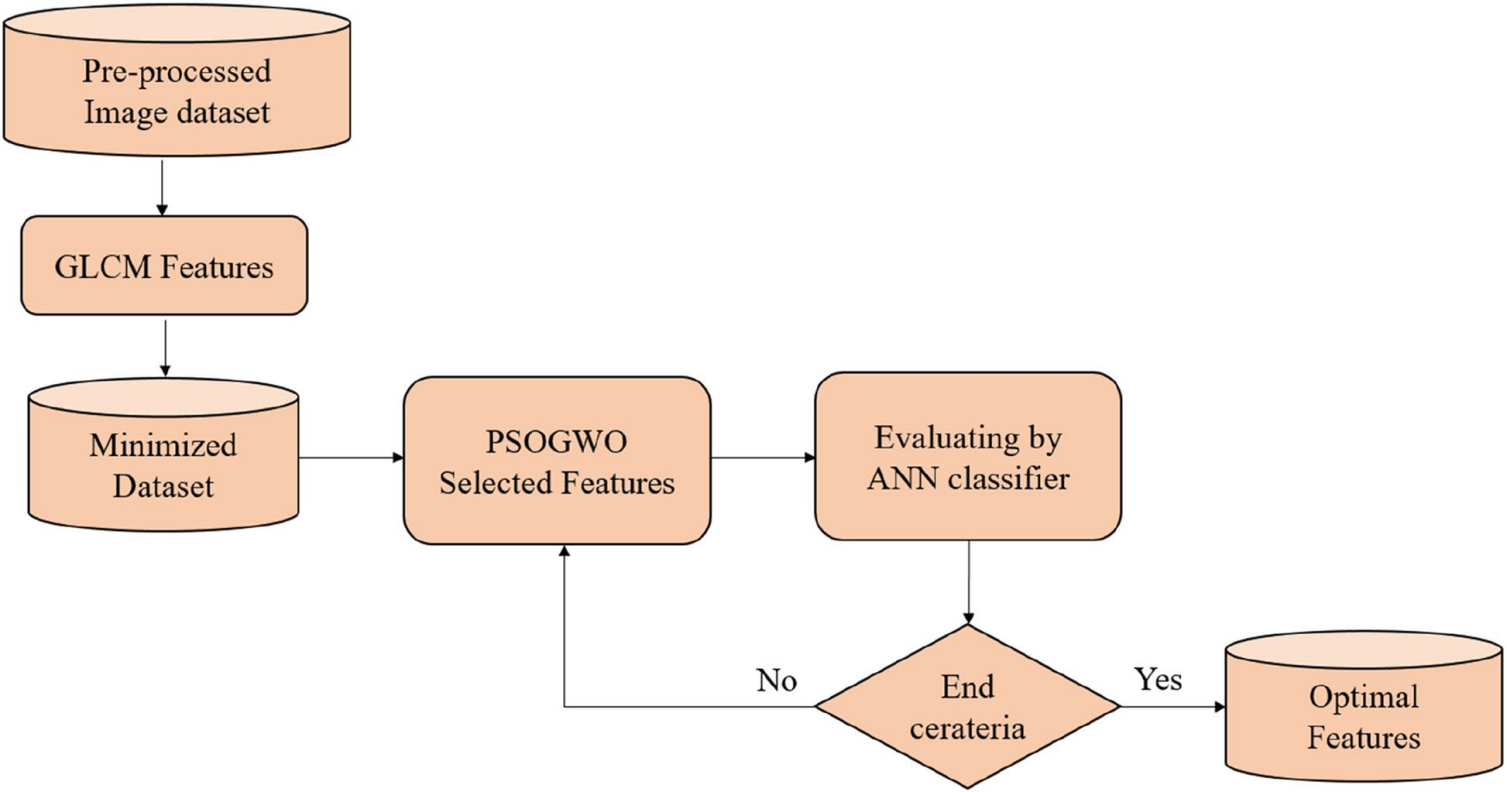
Working of PSOGWO algorithm for feature selection


Figure 4 depicts the overall structure of PSOGWO for optimized feature selection. PSOGWO combines PSO and GWO wrapping algorithms. At this point, testing the effectiveness of the two algorithms, GWO and PSO, which employ different search methodologies, is desired. When it comes to feature selection algorithms, PSO is a favorite among researchers. The PSO's update mechanism is integrated into the fundamental design of GWO. The first two stages of PSOGWO are parameterization and population formation, which cover a wide range of problem solutions (feature selection). Calculating the fitness function for each solution and selecting the best one helps us to assess their utility. The next stage of the PSOGWO algorithm involves updating the population with the concurrent GWO and PSO algorithms. Following that, the grey wolf fitness function is compared to the best in the world, and the variables are adjusted as needed. Equation (19) is utilized to determine the fitness function.

19 $$fitness={weight}_{acc}*accuracy\left(agent\right)+weigh{t}_{-}feature* \frac{to{t}_{-}feat-se{l}_{-}feat}{to{t}_{-}feat}$$

where $tot\_ feat$ is the total features in the agent, $sel\_ feat$ is the features selected by the agent, and $accuracy(agent)$ is the agent's classification accuracy. The parameters are then revised to reflect the new positions, and the grey wolves and global best adjust to match. Once the final requirements are met, the process is repeated. The ultimate result is a vector of integers indicating whether characteristics were selected or removed. As illustrated in Algorithm 3, the PSOGWO technique is recommended.

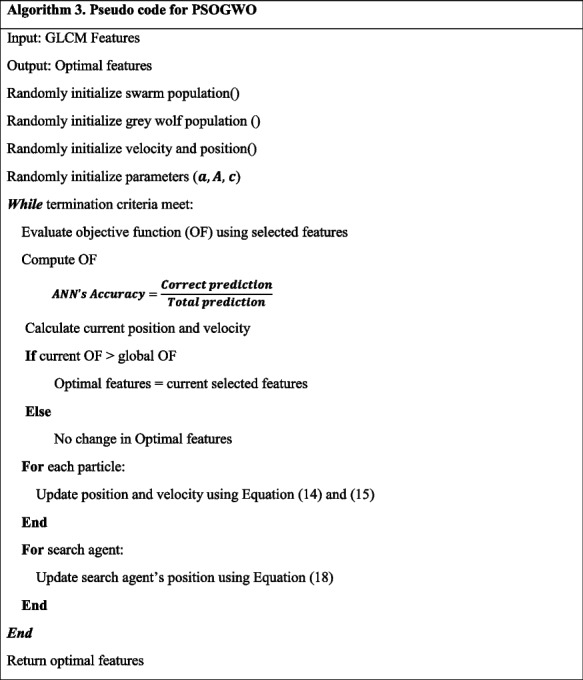


### Classifiers

**Fig. 5:**
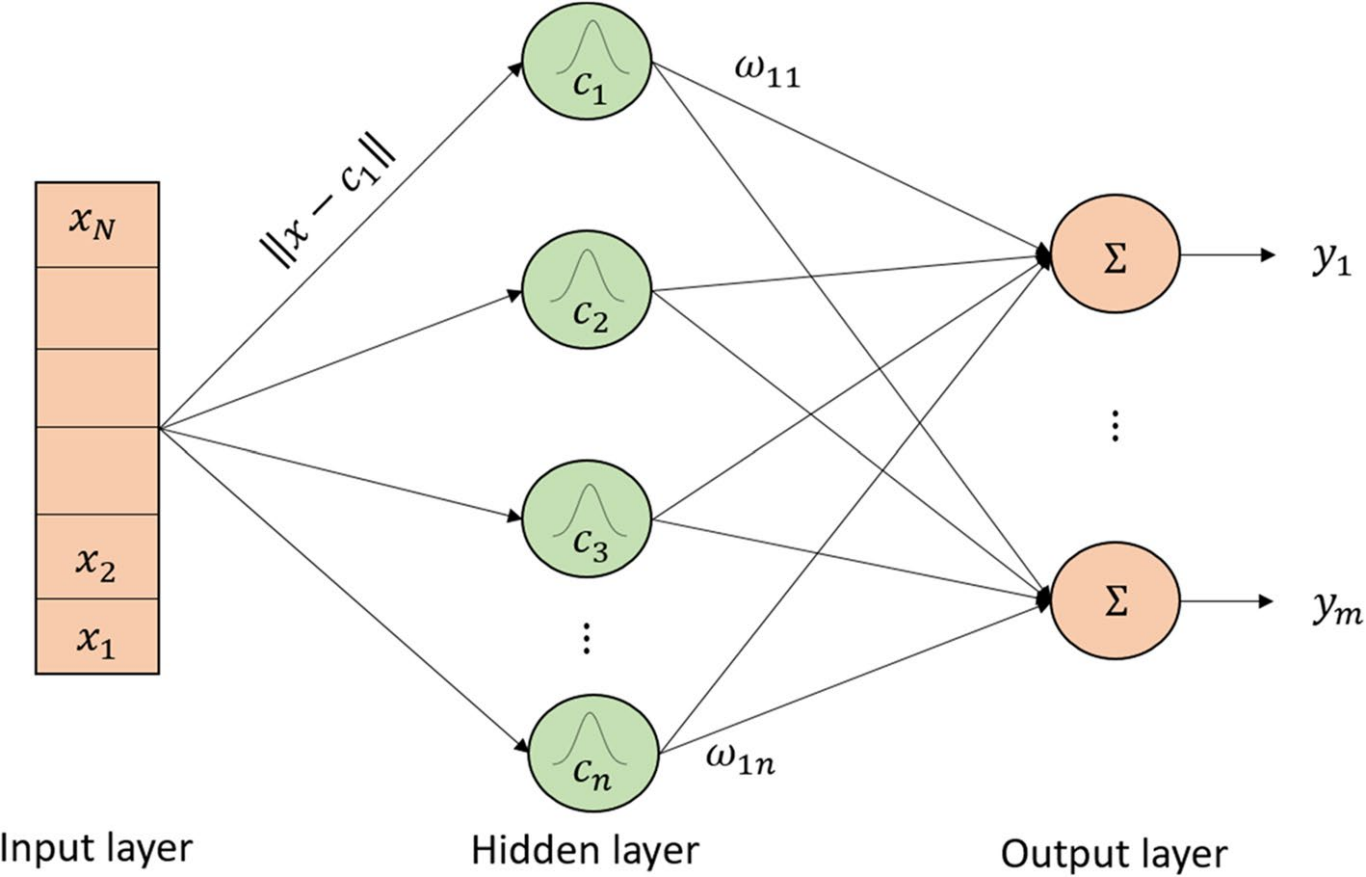
RBFNN Architecture

Figure 5 depicts the three layers that comprise RBFNN: Input Layer (IL), Hidden Layer (HL), and Output Layer (OL) [41]. This includes ${x}_{i}\in {R}^{d}$ and $y\in R$, with $M$ hidden layer nodes, and the RBFNN executes the nonlinear mapping $f:{R}^{d}\to R$. The training US images are fed into the RBFNN's input layer, while the HL nodes conduct a nonlinear transformation using the RBF for mapping the input and new space. When constructing a Gaussian function, the variables in the RBF reflect the centre $({c}_{i}\in {R}^{d})$ and kernel width (${\delta }_{i}$). The function is described in the Eq. (20):

20 $$\varphi \left(\Vert x-{c}_{i}\Vert \right)=exp\left(-\frac{{\Vert x-{c}_{i}\Vert }^{2}}{{\delta }_{i}}\right)$$

A linear weighted combination is applied in the new space by the OL’s nodes. For the mapping function from ${R}^{d}\to R$, let's assume *φ*(∎) is the RBF and ${w}_{i}$ is the connection weight between the HL and OL.

21 $$y=f\left(x\right)=\sum_{i=1}^{M}{w}_{i}\varphi \left(\Vert x-{c}_{i}\Vert \right)$$

With the use of a linear model and an RBFNN. As stated in the introduction, three parameters are needed for the RBFNN. The parameters are the centre vector of the RBFNN ${c}_{i}={\left[{c}_{i1},{c}_{i2},\dots ,{c}_{id}\right]}^{T}$, the width of the kernel ${\delta }_{i}$, and the weight of the OL ${w}_{i}$. Whereas ${w}_{i}$ and ${\delta }_{i}$ are determined by gradient descent learning [42] and fuzzy C-means (FCM) technique. Equations (22) and (23) represent the centre of the RBF $({c}_{ik})$ and the kernel width $({\delta }_{i})$, respectively.

22 $${c}_{ik}=\frac{{\sum }_{j=1}^{n}{\mu }_{ji}{x}_{jk}}{{\sum }_{j=1}^{n}{\mu }_{ji}}$$

23 $${\delta }_{i}=\frac{{\sum }_{j=1}^{n}{\mu }_{ji}{\Vert {x}_{j}-{c}_{i}\Vert }^{2}}{{\sum }_{j=1}^{n}{\mu }_{ji}}$$

24 $$\text{Let} \widetilde{{x}_{i}}=\varphi \left(\Vert x-{c}_{i}\Vert \right), i=\text{1,2},\dots ,M$$

25 $$\widetilde{x}={\left[{\widetilde{x}}_{1},{\widetilde{x}}_{2},\dots ,{\widetilde{x}}_{M}\right]}^{T}$$

where, n represents the training US image samples, $M$ represents the Total HL nodes, and ${\mu }_{ji}$ represents the outcome of FCM. The input sample is mapped to the new space $f:{R}^{d}\to {R}^{M}$, and the transformation from the IL to the HL creates a nonlinear mapping. The network operation is expressed in Eq. (26) and let's assume $p={\left[{w}_{1},{w}_{2},\dots {w}_{M}\right]}^{T}$.

26 $$y={p}^{T}\widetilde{x}$$

The network's output can be transformed into a linear model by calculating the RBF’s HL, as illustrated in Eq. (26).

#### Fast-RBFNN

The RBF linear model's ε-insensitive loss function (ILF) is introduced through the fast-RBF search principle. The ε-ILF refers to a loss function that reduces sensitivity to noise or variations in data, enabling more important features to be identified. Unlike common loss functions such as cross-entropy, which aim to minimize overall prediction error, this function prioritizes robustness, making it more suitable for medical imaging tasks. By determining the significance of the constraint term ε, the optimization seeks to minimize the ε-ILF. The RBFNN optimization model is constructed using the Gaussian kernel and incorporates the structural risk term with large-sample processing. The structural risk term acts as a regularization component in the loss function, controlling model complexity. It helps mitigate overfitting by penalizing excessively complex models, ensuring better generalization to new data, and enhancing overall performance. The processes are detailed below.

1. Equations (22) and (23) yield the values of ${c}_{i}$ and ${\delta }_{i}$, respectively, while Eq. (5) yields the model input $\widetilde{x}$.
2. The loss function unaffected by ε is presented. The definition of the ε-ILF is as follows:

27 $${L}^{\varepsilon}\left(x,y,f\right) = \left|y-f\left(x\right)\right|_{\varepsilon} = max\left(0,{\left|y-f\left(x\right)\right|}_{\varepsilon}\right),\, x\in {R}^{d},y\in R$$

Equation (26)'s linear model can have its equivalent ε-ILF written as

28 $$\sum_{i=1}^{n}{\left|{y}_{i}^{o}-{y}_{i}\right|}_{\varepsilon }=\sum_{i=1}^{n}max\left(0,\left|{y}_{i}^{o}-{y}_{i}\right|-\varepsilon \right)=\sum_{i=1}^{n}max\left(0,\left|{p}^{T}{\widetilde{x}}_{i}-{y}_{i}\right|-\varepsilon \right)$$

The output value of the neural network is denoted by ${y}_{i}^{o}$, while ${y}_{i}$ represents the actual output value. When the constraints of ${p}^{T}{\widetilde{x}}_{i}-{y}_{i}<\varepsilon$ and ${y}_{i}-{p}^{T}{\widetilde{x}}_{i}<\varepsilon$ are not always satisfied, the relaxation factors ${\xi }_{i}$ and ${\xi }_{i}^{*}$ are added.

29 $$\left\{\begin{array}{c}{y}_{i}-{p}^{T}{\widetilde{x}}_{i}<\varepsilon +{\xi }_{i}, {\xi }_{i}\ge 0\\ {p}^{T}{\widetilde{x}}_{i}-{y}_{i}<\varepsilon +{\xi }_{i}^{*}, {\xi }_{i}^{*}\ge 0\end{array}\right.$$

To minimize the value of the ε-ILF, represented by Eq. (8), this algorithm exists. The ε-insensitive parameter's value has a direct impact on the precision of the models. Therefore, the optimization problem incorporates the parameter λ and utilizes ε as the constraint term. When the optimization problem is coupled with Eq. (29), the resulting expression remains the same and it is represented in Eq. (27)

30 $$\text{min}2\lambda \varepsilon +\frac{\lambda }{\mu n}\sum_{i=1}^{n}\left({\xi }_{i}^{2},{\xi }_{i}^{{*}^{2}}\right), s.t\left\{\begin{array}{c}{y}_{i}-{p}^{T}{\widetilde{x}}_{i}<\varepsilon +{\xi }_{i}\\ {p}^{T}{\widetilde{x}}_{i}-{y}_{i}<\varepsilon + {\xi }_{i}^{*}\end{array}\right.$$

The condition that ${\xi }_{i},{\xi }_{i}^{*}\ge 0$ is automatically met, and $\mu$ is the equilibrium factor.

3. Introducing kernel functions and structural risk items: A support vector machine (SVM) is one method for minimizing structural risk [43, 44]. This work presents a strategy for learning how to design a SVM and incorporating a regularization to reduce algorithmic risk. SVMs use the kernel strategy to enhance the computational capabilities of linear learners. The proposed technique in this paper also involves the use of a kernel function [45]. Employing the regularization and kernel function can help to represent the optimization problem and it is given in Eq. (28).

31 $$\begin{array}{c}min\\ p,\varepsilon ,{\xi }_{i},{\xi }_{i}^{*}\end{array}{\Vert p\Vert }^{2}+2\lambda \varepsilon +\frac{\lambda }{\mu n}\sum_{i=1}^{n}\left({\xi }_{i}^{2}+{\xi }_{i}^{{*}^{2}}\right), s.t\left\{\begin{array}{c}{y}_{i}-{p}^{T}\varphi \left({\widetilde{x}}_{i}\right)<\varepsilon +{\xi }_{i},\\ {p}^{T}{\varphi \widetilde{x}}_{i}-{y}_{i}<\varepsilon + {\xi }_{i}^{*},\\ i=\text{1,2},..,n\end{array}\right.$$

4. Formula derivation: The Lagrangian function can be written as a function of time when the Lagrange multiplier is introduced.

32 $$L={\Vert p\Vert }^{2}+2\lambda \varepsilon +\frac{\lambda }{\mu n}\sum_{i=1}^{n}\left({\xi }_{i}^{2}+{\xi }_{i}^{{*}^{2}}\right)+\sum_{i=1}^{n}{\alpha }_{i}\left({y}_{i}-{p}^{T}\varphi \left({\widetilde{x}}_{i}\right)-\varepsilon -{\xi }_{i}\right)+\sum_{i=1}^{n}{\alpha }_{i}^{*}\left({p}^{T}\varphi \left({\widetilde{x}}_{i}\right)-{y}_{i}-\varepsilon -{\xi }_{i}^{*}\right)$$

The related dual problem of Eq. (29) has the following matrix form:

33 $$\left\{max\left[{\alpha }^{T}{\alpha }^{{*}^{T}}\right]\right.\left[\genfrac{}{}{0pt}{}{\frac{2}{\lambda }y}{-\frac{2}{\lambda }y}\right]-\left[{\alpha }^{T} {\alpha }^{{*}^{T}}\right]\widetilde{K}\left[\genfrac{}{}{0pt}{}{\alpha }{{\alpha }^{*}}\right], s.t \left[{\alpha }^{T} {\alpha }^{{*}^{T}}\right]1=1, \alpha ,{\alpha }^{*}\ge 0$$

where $\alpha ,{\alpha }^{*}$ and $\widetilde{K}$ represents Lagrange coefficients and kernel function.

34 $$y=\left[\begin{array}{c}{y}_{1}\\ \vdots \\ {y}_{n}\end{array}\right],\alpha =\left[\begin{array}{c}{\alpha }_{1}\\ \vdots \\ {\alpha }_{n}\end{array}\right],{\alpha }^{*}=\left[\begin{array}{c}{\alpha }_{1}^{*}\\ \vdots \\ {\alpha }_{n}^{*}\end{array}\right]$$

35 $$\widetilde{K}=\left[\widetilde{k}\left({\widetilde{x}}_{i},{\widetilde{x}}_{j}\right)\right]=\left[\begin{array}{cc}K+\frac{\mu n}{\lambda }I& -K\\ -K& K+\frac{\mu n}{\lambda }I\end{array}\right]$$

where $K$ represents the Gaussian kernel. The solution's variable values are

36 $$\left\{\begin{array}{c}p=\lambda \sum_{i=1}^{n}\left({\alpha }_{i}-{\alpha }_{i}^{*}\right)\varphi \left({\widetilde{x}}_{i}\right)\\ {\xi }_{i}={\alpha }_{i}\mu n\\ {\xi }_{i}^{*}={\alpha }_{i}^{*}\mu n.\end{array}\right.$$

5. Classification: The classifier function is illustrated in the Eq. (35)

37 $$y=sign\left({p}^{T}\varphi \left({\widetilde{x}}_{test}\right)\right)=\lambda \sum_{i=1}^{n}\left({\alpha }_{i}-{\alpha }_{i}^{*}\right){\varphi }^{T}\left({\widetilde{x}}_{i}\right)\varphi \left({\widetilde{x}}_{test}\right)=\lambda \sum_{i=1}^{n}\left({\alpha }_{i}-{\alpha }_{i}^{*}\right)\widetilde{K}\left({\widetilde{x}}_{i},{\widetilde{x}}_{test}\right)$$

A fetal brain, abdomen, femur, thorax, maternal cervix and other is associated with a y-value that falls anywhere from 0 to 5.

## Result and discussion

### Experimental setup

A novel methodology for identifying fetal organs in US images is introduced, utilizing the Zendo dataset. After pre-processing, features are extracted using GLCM techniques. These features are then inputted into conventional DL models, including ANN, CNN, RBF) and our proposed fast RBFNN. The experimental environment configuration setting used in this research is displayed in Table 4.

**Table 4:** Experimental environment

Model	Accuracy
Operating System	Windows 11
CPU	Intel(R) Xeon(R) Gold 6330
GPU	NVIDIA GeForce RTX
RAM	32 GB
Programming Language	Python
Environment	Anaconda – JupyterNotebeook
Proposed	96.37

### Evaluation metrics

Assessing DL model performance is critical, achieved through various metrics such as Accuracy, Specificity, Sensitivity, Precision, F1 score, and Matthews Correlation Coefficient (MCC), False Rejection Rate (FRR) and False Acceptance Rate (FAR). Table 5 details the formula for each metric. In these formulas, $TP$ represents True Positives, $TN$ represents True Negatives, $FP$ represents False Positives, and $FN$ represents False Negatives in the classification of fetal organs in US images.

**Table 5:** Evaluation metrics

Metrics	Formula
Accuracy	$\frac{TP+TN}{TP+TN+FP+FN}$
Specificity	$\frac{TN}{TN+FP}$
Sensitivity	$\frac{TP}{TP+FN}$
Precision	$\frac{TP}{TP+FP}$
F1 score	$\frac{TP.TN-FP.FN}{\sqrt{(TP+FP)(TP+FN)(TN+FP)(TN+FN)}}$
MCC	$\frac{TP}{TP+FN}$
FRR	$\frac{FN}{TP+FN}$
FAR	$\frac{FP}{TN+FP}$

### Experimental outcome

Table 6 gives the performance metrics attained by the convention and proposed DL models using GLCM features. Our proposed model exhibits the highest performance metrics, with Accuracy, Specificity, Sensitivity, Precision, F1 score, and MCC at 96.37%, 97.08%, 95.67%, 97.02%, 96.34%, and 92.75%, respectively, and minimal FRR and FAR of 4.33 and 2.92 using GLCM features.

**Table 6:** DL model performance on fetal US image classification using GLCM features

Model	Accuracy	Specificity	Sensitivity	Precision	F1	MCC	FRR	FAR
ANN	92.59	93.88	91.35	93.92	92.62	85.22	8.65	6.12
CNN	93.06	94.25	91.87	94.18	93.01	86.13	8.13	5.75
RBF	94.98	95.25	94.71	95.16	94.93	89.96	5.29	4.75
Proposed	96.37	97.08	95.67	97.02	96.34	92.75	4.33	2.92

To enhance model performance, essential features are selected using optimized algorithms like PSO, GWO, and a hybrid PSO-GWO approach. The performance of both conventional and proposed DL models, utilizing features selected by these algorithms, is presented in Tables 7, 8 and 9. The combination of hybrid PSOGWO-selected features with our proposed model yields the most satisfactory performance, achieving 98.07% Accuracy, 98.12% Specificity, 98.03% Sensitivity, 98.18% Precision, 98.10% F1 score, and 96.15% MCC.

**Table 7:** DL model performance on fetal US image classification using PSO selected features

Model	Accuracy	Specificity	Sensitivity	Precision	F1	MCC	FRR	FAR
ANN	94.06	94.70	93.44	94.74	94.08	88.12	6.56	5.30
CNN	96.06	97.06	95.08	97.02	96.04	92.14	4.92	2.94
RBF	96.84	97.68	96.00	97.65	96.82	93.68	4.00	2.32
Proposed	97.54	98.31	97.37	98.28	97.83	95.68	2.63	1.69

**Table 8:** DL model performance on fetal US image classification using GWO selected features

Model	Accuracy	Specificity	Sensitivity	Precision	F1	MCC	FRR	FAR
ANN	94.91	95.52	94.29	95.47	94.88	89.82	5.71	4.48
CNN	95.22	96.39	94.08	96.42	95.24	90.46	5.92	3.81
RBF	96.76	97.23	96.29	97.19	96.74	93.52	3.71	2.77
Proposed	97.84	98.16	97.52	98.13	97.82	95.68	2.48	1.84

**Table 9:** DL model performance on fetal US image classification using Hybrid PSOGWO selected features

Model	Accuracy	Specificity	Sensitivity	Precision	F1	MCC	FRR	FAR
ANN	95.52	96.43	94.63	96.41	95.51	91.06	5.37	3.57
CNN	96.53	96.19	96.87	96.12	96.49	93.05	3.13	2.05
RBF	97.07	97.68	96.46	97.66	97.05	94.14	3.54	2.32
Proposed	98.07	98.12	98.03	98.18	98.10	96.15	1.97	1.88

**Fig. 6:**
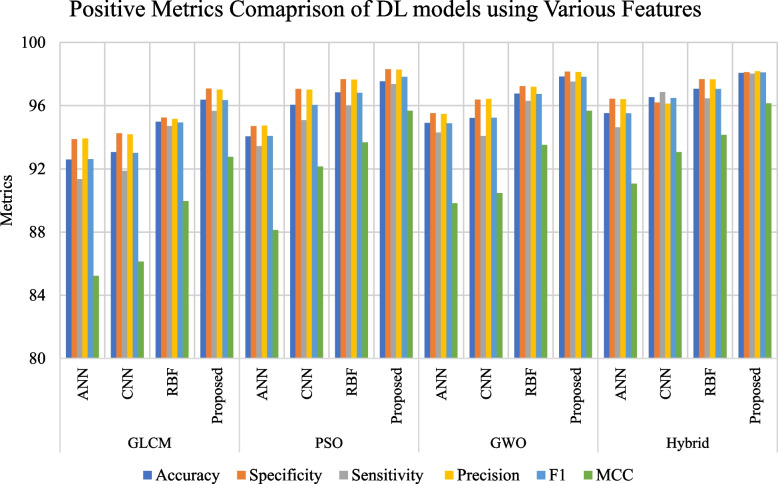
Evaluation of the DL model’s positive metrics by employing various features

**Fig. 7:**
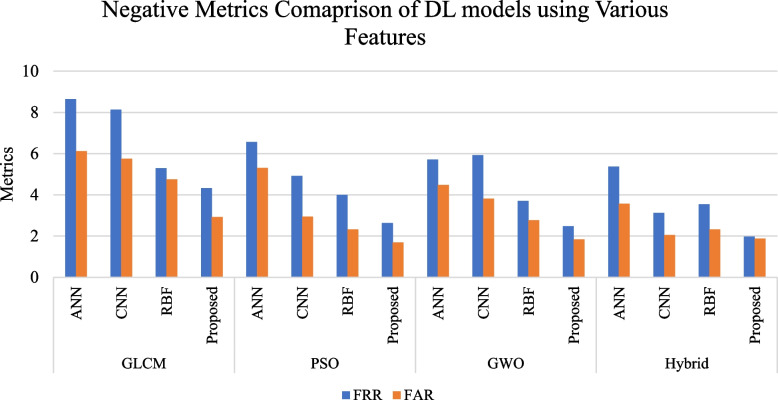
Evaluation of the DL model’s negative metrics by employing various features

Figures 6 and 7 illustrate a comparison of DL models based on positive and negative metrics using various feature extraction techniques, including GLCM, PSO, GWO, and hybrid approaches. The figures highlight that the proposed model, which utilizes hybrid features, outperforms other DL models and feature extraction techniques in terms of both positive and negative metrics. This demonstrates the superior performance and effectiveness of the proposed framework.

The speckle noise removal is an important pre-processing step. The outcome of the DL models using Hybrid PSOGWO-selected features gives better results in fetal organ classification. To demonstrate the importance of the pre-processing step, the DL model is evaluated using the Hybrid PSOGWO-selected features with the input image containing speckle noise. The outcome of the DL model using Hybrid PSOGWO-selected features without noise removal is presented in Table 10. By comparing Tables 9 and 10, the DL model achieves the highest positive metrics and lower negative metrics on input images without noise.

**Table 10:** DL model performance on fetal US image classification using Hybrid PSOGWO selected features without noise removal

Model	Accuracy	Specificity	Sensitivity	Precision	F1	MCC	FRR	FAR
ANN	94.47	95.24	93.25	95.47	94.35	90.01	6.24	4.83
CNN	95.23	95.03	95.65	94.79	95.22	92.55	3.98	3.62
RBF	95.89	96.14	95.98	96.24	96.11	93.69	4.57	3.46
Proposed	96.54	97.2	96.89	96.88	96.88	95.78	2.68	2.49

The performance of the proposed model in classifying each organ from US images is evaluated, with results summarized in Table 11. The proposed model exhibits high accuracy across various organs: 97.69% for the brain, 98.15% for the abdomen, 99.07% for the femur, 97.69% for the thorax, 97.22% for maternal cervix, and 98.61% for other organs. In addition to accuracy, other metrics for each organ are also provided in Table 11, offering a comprehensive assessment of the proposed model's performance across different organs in fetal imaging.

**Table 11:** Evaluation of the proposed methodology for each image classification

	Accuracy	Specificity	Sensitivity	Precision	F1	MCC	FRR	FAR
Brain	97.69	98.10	97.30	98.18	97.74	95.37	2.70	1.90
Abdomen	98.15	99.04	97.32	99.09	98.20	96.31	2.68	0.96
Femur	99.07	99.06	99.09	99.09	99.09	98.14	0.91	0.94
Thorax	97.69	97.20	98.17	97.27	97.72	95.37	1.83	2.80
Maternal Cervix	97.22	96.30	98.15	96.36	97.25	94.46	1.85	3.70
Other	98.61	99.06	98.18	99.08	98.63	97.22	1.82	0.94
Average	98.07	98.12	98.03	98.18	98.10	96.15	1.97	1.88

The time efficiency of each stage of the proposed methodology on test data is evaluated and presented in Table 12. Pre-processing and feature extraction using GLCM require 4 s and 11 s, respectively. Subsequently, feature selection using the hybrid PSO-GWO algorithm consumes a significant time of 5 min and 29 s. For classification, ANN takes 38 s, while our proposed model takes 57 s, although it is faster than CNN and RBF models. This assessment provides insight into the computational time required by the proposed method. Overall, the finalized methodology, comprising pre-processing, GLCM feature extraction, PSOGWO-based feature selection, and fast RBF classification, takes 6 min and 41 s to process the test data.

**Table 12:** Analysis of operational efficiency on test data

Steps	Method	Time-Consuming
Pre-processing	-	4 s
Feature Extraction	GLCM	11 s
Feature Selection	PSO	2 min 20 s
	GWO	3 min 12 s
	PSOGWO	5 min 29 s
Classifiers	ANN	38 s
	CNN	1 min 5 s
	RBF	1 min 12 s
	Proposed	57 s

In assessing the effectiveness of the proposed model, a comparative analysis with existing models from recent research was conducted, as presented in Table 13. Analysis revealed that several references, namely [46, 48], and [49], evaluated their models solely based on accuracy, without considering other essential metrics. In contrast, our proposed model offers comprehensive metrics. The highest accuracy reported in previous research is 96.85% and 96% from studies [47, 48]. The proposed model achieves 98.07%, surpassing the existing best accuracy. For sensitivity and precision, the highest scores reported are from references [24, 47], with sensitivity values of 96.28% and 96.66%, and precision values of 94.02% and 97.12%. The proposed model yields 98.12% sensitivity and 98.18% precision, which is significantly better than the existing results. The MCC values reported in research [19, 24] are 74.8% and 94.19%, whereas the proposed model achieves an MCC of 96.15%. Additionally, for the F1 score, the proposed model outperforms existing values, which range from 79 to 97%. Overall, the comparative analysis highlights the effectiveness and superiority of our proposed model over existing approaches, showcasing its potential for advancing fetal organ identification in US imaging research.

**Table 13:** Comparison of the proposed model with existing works

Ref	Accuracy	Sensitivity	Precision	MCC	F1 score
[19]	79.4	79.45	78.95	74.8	79.1
[46]	93.6	-	-	-	-
[24]	95.69	96.28	94.02	94.19	95.08
[47]	96.85	96.66	97.12	-	96.88
[48]	96	-	-	-	-
[49]	94				
Our	98.07	98.12	98.18	96.15	98.10

### Discussion

The proposed model, using hybrid PSO-GWO features, delivers excellent results and outperforms existing models such as ANN, CNN, and RBF. The model achieves accuracy, sensitivity, precision, MCC, and F1 scores of 98.07%, 98.12%, 98.18%, 96.15%, and 98.10%, respectively. Table 13 demonstrates that the proposed model also performs better than existing research. The ε-insensitive loss function (ε-ILF) and advanced feature selection contribute to the model's improved performance by handling noisy images more effectively. Additionally, structural risk minimization helps reduce overfitting. Another reason for the improved results is the selection of more suitable features using the hybrid PSO-GWO approach. Table 11 presents the model’s performance in classifying each organ, indicating that the model accurately identifies all organs. The outcomes of the proposed framework suggest that the model is suitable for real-time deployment and provides promising results in identifying fetal organs from US images.

However, the proposed framework also presents challenges. The execution time for feature extraction, feature selection using the hybrid model, and classification is 11 s, 5 min 29 s, and 57 s, respectively. The complexity of the framework increases the execution time. Analysis of Tables 9 and 10, which compare the hybrid DL model with and without noisy images, reveals that the model performs better without noisy images. This indicates that the proposed model still relies heavily on pre-processing steps to achieve optimal performance.

## Conclusion

The research successfully designs a novel methodology using hybrid optimized feature selection and the Fast RBFNN model for maternal fetal US plane classification. US images are the safest technique for fetal and maternal health monitoring. These images are processed and features are extracted using the GLCM technique. The most important features are selected by the PSOGWO algorithm, as well as PSO and GWO. The selected features from each algorithm are then fed into DL classifiers such as ANN, CNN, RBF, and Fast RBFNN. All combinations of output features from GLCM, PSO, GWO, and PSOGWO with DL models are tested. Experimental results show that the proposed model performs better on hybrid PSOGWO features. The accuracy attained by the proposed model is 98.07%, 97.84%, 97.54%, and 96.37% on PSOGWO, GWO, PSO, and GLCM features, respectively. Next, the model efficiency is compared using time consumption. The proposed DL model takes only 57 s to classify the test data, indicating its suitability for real-time applications. A limitation of the research is the use of data from the same dataset for both training and testing. To better determine the model's effectiveness, it is necessary to compare it with additional datasets. Future work will involve evaluating the model with diverse datasets and exploring its deployment for clinical use in real-time applications.

## Data Availability

No datasets were generated or analysed during the current study.
